# Docosahexaenoic acid supplementation represses the early immune response against murine cytomegalovirus but enhances NK cell effector function

**DOI:** 10.1186/s12865-022-00492-6

**Published:** 2022-04-19

**Authors:** Shuting Wu, Shanshan Wang, Lili Wang, Hongyan Peng, Shuju Zhang, Qinglan Yang, Minghui Huang, Yana Li, Shuzhen Guan, Wenjuan Jiang, Zhaohui Zhang, Qinghua Bi, Liping Li, Yuan Gao, Peiwen Xiong, Zhaoyang Zhong, Bo Xu, Yafei Deng, Youcai Deng

**Affiliations:** 1 Pediatrics Research Institute of Hunan Province, Hunan Children’s Hospital, Changsha, Hunan People’s Republic of China; 2 Pediatric Intensive Care Unit, Hunan Children’s Hospital, University of South China, Changsha, Hunan People’s Republic of China; 3 State Key Laboratory of Ophthalmology, Zhongshan Ophthalmic Center, Sun Yat-Sen University, Guangzhou, 510060 People’s Republic of China; 4 Institute of Materia Medica, College of Pharmacy, Army Medical University (Third Military Medical University), Chongqing, 400038 People’s Republic of China; 5 Southwest Hospital/Southwest Eye Hospital, Army Medical University (Third Military Medical University), Chongqing, 400038 People’s Republic of China; 6 Cancer Center, Daping Hospital and Research Institute of Surgery, Army Medical University (Third Military Medical University), Chongqing, 400042 People’s Republic of China; 7 Jiangsu Center for the Collaboration and Innovation of Cancer Biotherapy, Cancer Institute, Xuzhou Medical University, Xuzhou, 221002 People’s Republic of China

**Keywords:** Docosahexaenoic acid, Cytomegalovirus, Natural killer cell, Mitochondrial activity, T cell activation

## Abstract

**Background:**

Docosahexaenoic acid (DHA) supplementation is beneficial for several chronic diseases; however, its effect on immune regulation is still debated. Given the prevalence of cytomegalovirus (CMV) infection and because natural killer (NK) cells are a component of innate immunity critical for controlling CMV infection, the current study explored the effect of a DHA-enriched diet on susceptibility to murine (M) CMV infection and the NK cell effector response to MCMV infection.

**Results:**

Male C57BL/6 mice fed a control or DHA-enriched diet for 3 weeks were infected with MCMV and sacrificed at the indicated time points postinfection. Compared with control mice, DHA-fed mice had higher liver and spleen viral loads at day 7 postinfection, but final MCMV clearance was not affected. The total numbers of NK cells and their terminal mature cell subset (KLRG1^+^ and Ly49H^+^ NK cells) were reduced compared with those in control mice at day 7 postinfection but not day 21. DHA feeding resulted in higher IFN-γ and granzyme B expression in splenic NK cells at day 7 postinfection. A mechanistic analysis showed that the splenic NK cells of DHA-fed mice had enhanced glucose uptake, increased CD71 and CD98 expression, and higher mitochondrial mass than control mice. In addition, DHA-fed mice showed reductions in the total numbers and activation levels of CD4^+^ and CD8^+^ T cells.

**Conclusions:**

These results suggest that DHA supplementation represses the early response to CMV infection but preserves NK cell effector functions by improving mitochondrial activity, which may play critical roles in subsequent MCMV clearance.

**Supplementary Information:**

The online version contains supplementary material available at 10.1186/s12865-022-00492-6.

## Background

Dietary ω-3 polyunsaturated fatty acids (PUFAs) are abundant in nature and belong to a category of safety supplements that have been linked to a reduced risk for chronic diseases, such as cardiovascular diseases [1], cognitive decline [2] and cancer [3]. ω-3 PUFA supplementation during pregnancy also reduces the risk for premature birth and perinatal death and improves birth weight and neonatal growth and development [4, 5]. Due to these health benefits, ω-3 PUFA supplementation for the prevention of several diseases is increasing [6, 7]. However, the public is still confused about the benefit of ω-3 PUFAs due to contradictory findings regarding their immunosuppressive effects on systems targeting viral or bacterial infections. For example, dietary supplementation with fish oil, enriched in ω-3 PUFAs, has been shown to impair host resistance to *Mycobacterium tuberculosis* [8] and influenza in mice [9]. However, the lungs of mice whose diets were supplemented with only docosahexaenoic acid (DHA) and eicosapentaenoic acid (EPA), two main ω-3 PUFAs, had a lower *M. tuberculosis* bacterial load than those of controls [10, 11]. DHA-derived lipids, such as protectin D1 and protectin D1 isomers, have also been shown to suppress influenza virus replication and promote inflammation resolution [12]. This evidence suggests multifaceted roles for ω-3 PUFAs in the immune response against bacterial or viral infection, warranting further in-depth study.

Cytomegalovirus (CMV), a member of the herpesvirus family, is a widespread virus to which approximately 45–100% of the global population has been exposed [13]. Notably, CMV infection is the most common and serious opportunistic infection in individuals with human immunodeficiency virus infection or patients after hematopoietic stem cell or solid organ transplantation [14]. Congenital CMV infection is the most common viral infection in humans and is a leading cause of neurologic disabilities and hearing loss in children worldwide [15]. Natural killer (NK) cells are a vital component of innate immunity and play critical roles in controlling CMV viral replication; experimental depletion of NK cells leads to unchecked viral replication and increased mortality [16]. In C57BL/6 mice, NK cells undergo a nonselective phase mediated by proinflammatory cytokines and a specific phase driven by signaling via Ly49H, an NK cell activation receptor that can directly recognize the MCMV (Smith and K181)-encoded protein m157 expressed on infected cells. The recognition of Ly49H by its ligand, m157, results in the robust expansion of Ly49H^+^ NK cells and the persistent elevation of KLRG1, important for NK cell-mediated clearance of MCMV-infected cells in C57BL/6 mice [17]. NK cells also play critical roles in regulating steps of the adaptive immune response, such as T cell activation [18]. However, the effects of DHA supplementation on resistance against MCMV infection and the NK cell response remain largely unknown.

In the current study, we explored the susceptibility of C57BL/6 mice fed a DHA-enriched diet for 3 weeks to MCMV infection and assessed the numbers and maturation of NK cells in each tissue. We also explored the expression of molecules related to NK cell effector function and mitochondrial activity. Here, we show that DHA supplementation led to a reduced response to MCMV at the early but not later stage after infection. In addition, DHA supplementation preserved NK cell effector functions by improving metabolic status and mitochondrial activity, which may play critical roles in MCMV clearance in the later stage of infection.

## Results

### DHA supplementation inhibited the early but not late response to MCMV infection

**Fig. 1:**
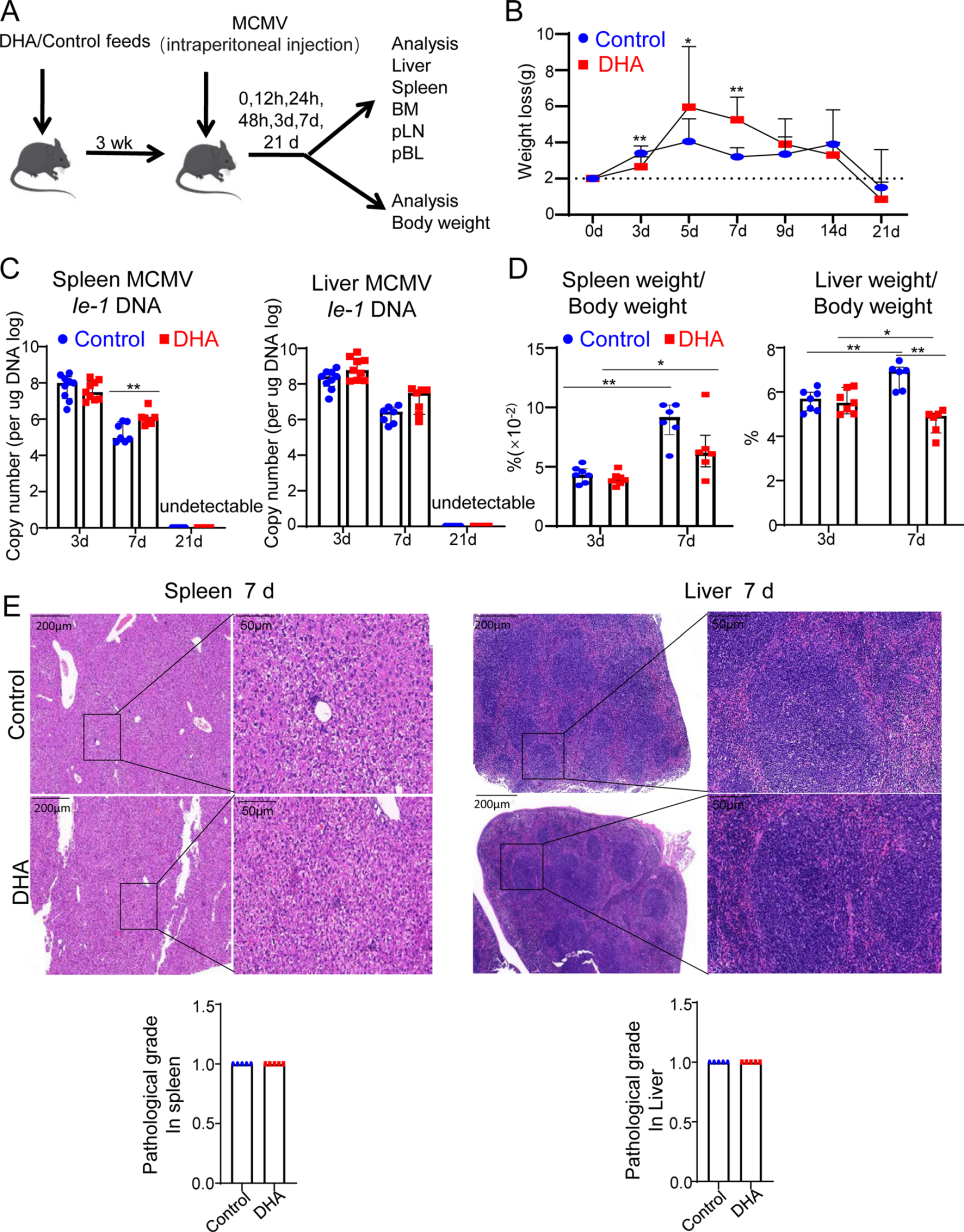
The effect of DHA feeding on MCMV resistance in C57BL/6 J mice. **A** Schematic diagram of the experimental design. **B** Weight loss change in mice fed a DHA-supplemented or control diet at days 0, 3, 5, 7, 9, 14, and 21 after MCMV infection. n = 6, pooled data of 2 independent experiments. **C** The MCMV *Ie-1* DNA level in the spleen (left) and liver (right) at days 3, 7, and 21 after MCMV infection was detected by qPCR, n = 9 at day 3; n = 7 at day 7; n = 6 at day 21. These data were pooled from 2–3 independent experiments. **D** The tissue/body weight ratio was calculated after weighing the spleen (left) and liver (right) tissues of mice fed a DHA-supplemented or control diet at days 3 and 7 following MCMV infection, n = 7 at day 3; n = 6 at day 7 postinfection. These are pooled data of 2 independent experiments. **E** H&E staining of the spleen and liver tissue at day 7 post MCMV infection. Histopathological quantification of inflammation in the spleen and liver of control and DHA-fed mice is shown in the lower panel. n = 5 pooled data of 2 independent experiments. Each symbol represents an individual mouse. All experiments were replicated at least 2 times. Error bars represent interquartile ranges *p < 0.05; **p < 0.01

Five-week-old C57BL/6 mice were first fed a special DHA-enriched or control diet for 3 weeks to explore the effects of DHA on the susceptibility of mice to MCMV infection. Subsequently, the mice were challenged with 3 × 10^4^ PFU of MCMV in 200 μL of PBS by intraperitoneal injection and sacrificed at days 3, 7 or 21 (Fig. 1A). Weight loss, the virus replication level, the visceral coefficient, and the local tissue mRNA levels of inflammatory cytokines were determined at the indicated time postinfection as the main markers of illness severity following MCMV infection [19, 20]. We found that body weight loss was significantly lower at day 3 but higher at days 5 and 7 after MCMV infection in DHA-fed mice than in control mice. However, there was no significant difference in body weight loss between control and DHA-fed mice at days 9, 14 and 21 after MCMV infection (Fig. 1B).

The spleen, liver and salivary gland are the main target organs of MCMV after intraperitoneal infection [21], and our data showed that the levels of MCMV *Ie-1* DNA were increased in the spleen at day 7 postinfection but not day 3. The levels of MCMV *Ie-1* DNA were slightly increased in liver tissues at day 3 and 7 postinfection, but without statistical significance. At the dose of MCMV given, no MCMV *Ie-1* DNA was detected at day 21 postinfection in the spleen or liver in either the control or DHA-fed mice (Fig. 1C). Previous studies have revealed that MCMV exhibits protracted replication in salivary glands after acute infection, which peaks at day 14 postinfection in C57BL/6 mice [22]. To this end, we also determined the viral load in the salivary glands at day 14 postinfection. The data showed that the viral loads of the salivary glands were comparable between DHA-fed and control mice (Additional file 1: Fig. S1).

Viral infection is often accompanied by hepatosplenomegaly [23, 24]. Regarding the spleen/body weight and liver/body weight index, neither the spleen/body weight nor the liver/body weight was significantly different between control and DHA-fed mice at day 3 postinfection, but liver/body weight was significantly lower, and spleen/body weight was decreased, in DHA-fed mice compared with control mice at day 7 postinfection (Fig. 1D). Interestingly, in control mice, both spleen/body weight and liver/body weight index were significantly increased at day 7 postinfection compared with day 3 postinfection. When these two indexes at days 3 and 7 postinfection in DHA-fed mice were compared, neither index was dramatically increased in DHA-fed mice compared with control mice (Fig. 1D). H&E staining showed that the pathology of both the spleen and liver was not obviously different between control and DHA-fed mice at day 7 postinfection. We also calculated the pathological scores of the H&E staining for both the spleen and liver, as described by Quatrini et al. [25]. The data showed that the pathological scores of both the spleen and liver showed no significant difference between DHA-fed and control mice (Fig. 1E).

These findings demonstrated that DHA supplementation moderately impaired the early response to MCMV infection without affecting final MCMV clearance at the later stage.

### DHA feeding affected NK cell frequency, maturation and selective Ly49H expansion in the response to MCMV infection

**Fig. 2:**
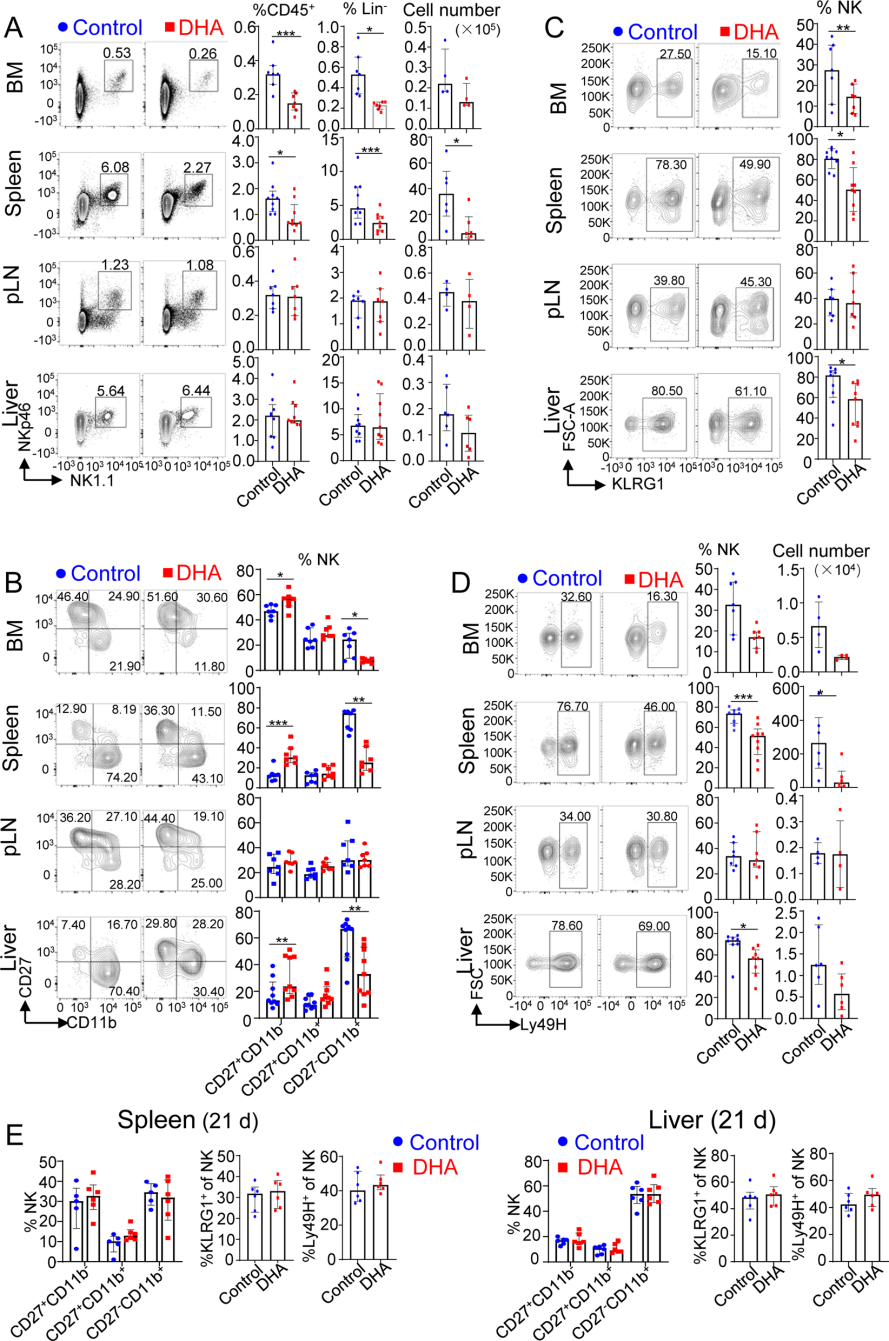
DHA feeding affected NK cell frequency, maturation and Ly49H^+^ NK cell expansion during the response to MCMV infection. **A** Flow cytometric analysis and enumeration of NK cells (CD45^+^CD3^−^CD19^−^ NK1.1^+^NKp46^+^) in the BM, spleens, pLNs and livers of control versus DHA-fed mice. **B** Flow cytometric analysis and cumulative frequencies of subpopulations of NK cell (CD3^−^CD19^−^NK1.1^+^NKp46^+^) subsets based on CD11b and CD27 expression in the BM, spleens, pLNs and livers of control versus DHA-fed mice. **C** Flow cytometric analysis of the KLRG1^+^ subsets of NK cells (CD3^−^CD19^−^NK1.1^+^NKp46^+^) in the BM, spleens, pLNs and livers of control versus DHA-fed mice at day 7 postinfection. **D** Flow cytometric analysis and enumeration of Ly49H^+^ subsets of NK cells (CD3^−^CD19^−^NK1.1^+^NKp46^+^) in the BM, spleens, pLNs and livers of control versus DHA-fed mice at day 7 postinfection. **E** Cumulative frequencies of NK cell (CD3^−^CD19^−^NK1.1^+^NKp46^+^) subsets found in the spleens, and livers of control versus DHA-fed mice at day 21 postinfection. For each experiment, n = 4 to 9 pooled from 2–3 independent experiments (**A**–**E**). Each symbol represents an individual mouse, and the blue dots and red square represent control and DHA-enriched diet-fed mice, respectively. Error bars represent interquartile ranges; *p < 0.05; **p < 0.01; ***p < 0.001

As the NK cell number peaks at day 7 after MCMV infection [26], we next explored the homeostasis and maturation of NK cells at day 7 after MCMV infection. We analyzed the proportion and numbers of NK cells in the bone marrow (BM), spleen, peripheral lymph nodes (pLNs), and liver tissue. Compared with those in control mice, the proportion and total numbers of NK cells (CD3^−^CD19^−^NK1.1^+^NKp46^+^ among CD45^+^ cells) were significantly decreased in the spleen but not other organs or tissues, although a similar trend without statistical significance was observed in the BM (Fig. 2A).

NK cells undergo accelerated phenotypic maturation in response to MCMV infection [26]. When the maturation of NK cells was investigated based on the expression of CD27 and CD11b [27], terminally matured NK cell (CD27^−^CD11b^+^) numbers were significantly reduced in the BM, spleen and liver, whereas immature NK cells (CD27^+^CD11b^−^) numbers were significantly increased in the spleen and liver of DHA-fed mice compared with control mice (Fig. 2B). We also determined the expression levels of KLRG1, another marker of NK cell terminal maturation [28], and found that it was also elevated on NK cells after MCMV infection [26]. Our data showed that compared with control mice, DHA-fed mice exhibited significantly reduced KLRG1^+^ NK cells in the BM, spleen and liver (Fig. 2C). Regarding the specific phase driven by Ly49H recognition [17], our data showed that DHA-fed mice had a significantly reduced ratio and total number of Ly49H^+^ NK cells in the spleen compared with those in control mice (Fig. 2D). However, there was no significant difference in the expression of CD11b, 27, KLRG1 or Ly49H at day 21 postinfection between control and DHA-fed mice (Fig. 2E).

As previous study revealed that daily consumption of 1 g ω-3 PUFA for 6 months resulted in reduced CD3^−^CD16^+^CD56^+^ NK cell numbers in human peripheral blood (pBL) leukocytes [29], we also determined the frequency, maturation and Ly49H expression levels of NK cells in the pBL of both DHA-fed and control mice the day before MCMV infection. The data revealed that 3 weeks of DHA feeding resulted in a reduced frequency of NK cells but without a significant effect on NK cell maturation or the frequency of Ly49H^+^ NK cells in the pBL (Additional file 1: Fig. S2).

Collectively, these data revealed that DHA supplementation reduced NK cell frequency but had no effect on NK cell maturation at a steady state. During MCMV infection, DHA feeding repressed NK cell maturation and Ly49H^+^ NK cell expansion in the main organs targeted by MCMV, such as the spleen and liver, at the early stage but not the later stage of MCMV infection.

### DHA-fed mice showed an enhanced capacity for IFN-γ production of NK cells and enhanced NK cell cytotoxicity during MCMV infection

**Fig. 3:**
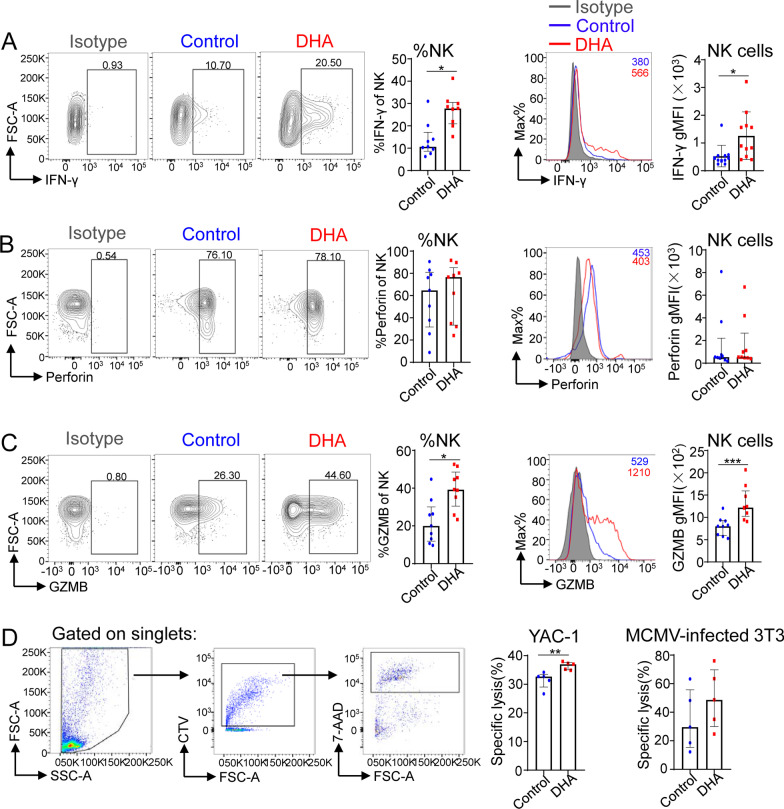
DHA feeding improved IFN-γ production and degranulation by NK cells in the spleen at day 7 postinfection. **A** Flow cytometric analysis of the ratio and the geometric mean fluorescence intensity (gMFI) of IFN-γ^+^ splenic NK cells (CD3^−^CD19^−^NK1.1^+^NKp46^+^) from control versus DHA-fed mice following stimulation with PMA and ionomycin in the presence of GolgiPlug and GolgiStop for 6 h. **B**, **C** Flow cytometric analysis depicting the frequencies and gMFI of perforin (**B**) and granzyme B (GZMB) (**C**) by splenic NK cells (CD3^−^CD19^−^NK1.1^+^NKp46^+^) from control versus DHA-fed mice at day 7 postinfection. **D** Purified splenic NK cells from both DHA-fed and control mice at day 7 after MCMV infection were cocultured with CTV-labeled YAC-1 (left) or MCMV-infected 3T3 cells (right) for 6 h at the E:T ratio of 16:1. Representative flow cytometry plots of CTV^+^ singlets are shown on the left. The percentages of NK cell-specific lysis are shown on the right, which were calculated by the following formula: [(% CTV ^+^ 7-AAD^+^ cell specific lysis − %CTV ^+^ 7-AAD^+^ cell spontaneous lysis)/ (100 − % CTV ^+^ 7-AAD^+^cell spontaneous lysis)] × 100. For each experiment, n = 9 pooled from 3 independent experiments (**A**–**C**); n = 5 pooled from 2 independent experiments (**D**). Each symbol represents an individual mouse, and the blue dots and red square represent control and DHA-enriched diet-fed mice, respectively. All experiments were replicated 3 times. Error bars represent interquartile ranges; *p < 0.05; **p < 0.01

Early during the course of infection, NK cells exert antiviral effects through direct toxicity and secretion of IFN-γ [30, 31]. Therefore, we next tested the ratio of IFN-γ^+^ NK cells after stimulation in vitro with PMA and ionomycin and determined the levels of the NK cell degranulation-related molecules perforin and granzyme B (GZMB) [32]. Our data revealed that the overall proportions and geometric mean fluorescence intensity (gMFI) of IFN-γ-secreting NK cells were increased in DHA-fed mice compared with control mice (Fig. 3A). The overall proportions and gMFI of GZMB, but not perforin, among splenic total NK cells were also increased in DHA-fed mice compared with control mice without any stimulation (Fig. 3B, C).

To further determine whether DHA feeding affects NK cell direct cytotoxicity post-MCMV infection, we sorted splenic NK cells from both DHA fed/unfed mice at day 7 after MCMV infection, and cocultured them with YAC-1 or MCMV-infected NIH-3T3 cells, followed by assaying the apoptosis of target cells. The data showed that the direct cytotoxicity of NK cells against both YAC-1 and MCMV-infected NIH-3T3 cells was enhanced in NK cells from DHA-fed mice, although it only showed statistical significance against YAC-1 (Fig. 3D).

These data suggest that DHA may improve NK cell effector function during MCMV infection.

### DHA feeding improved the cellular metabolic status and mitochondrial activity of NK cells during MCMV infection

**Fig. 4:**
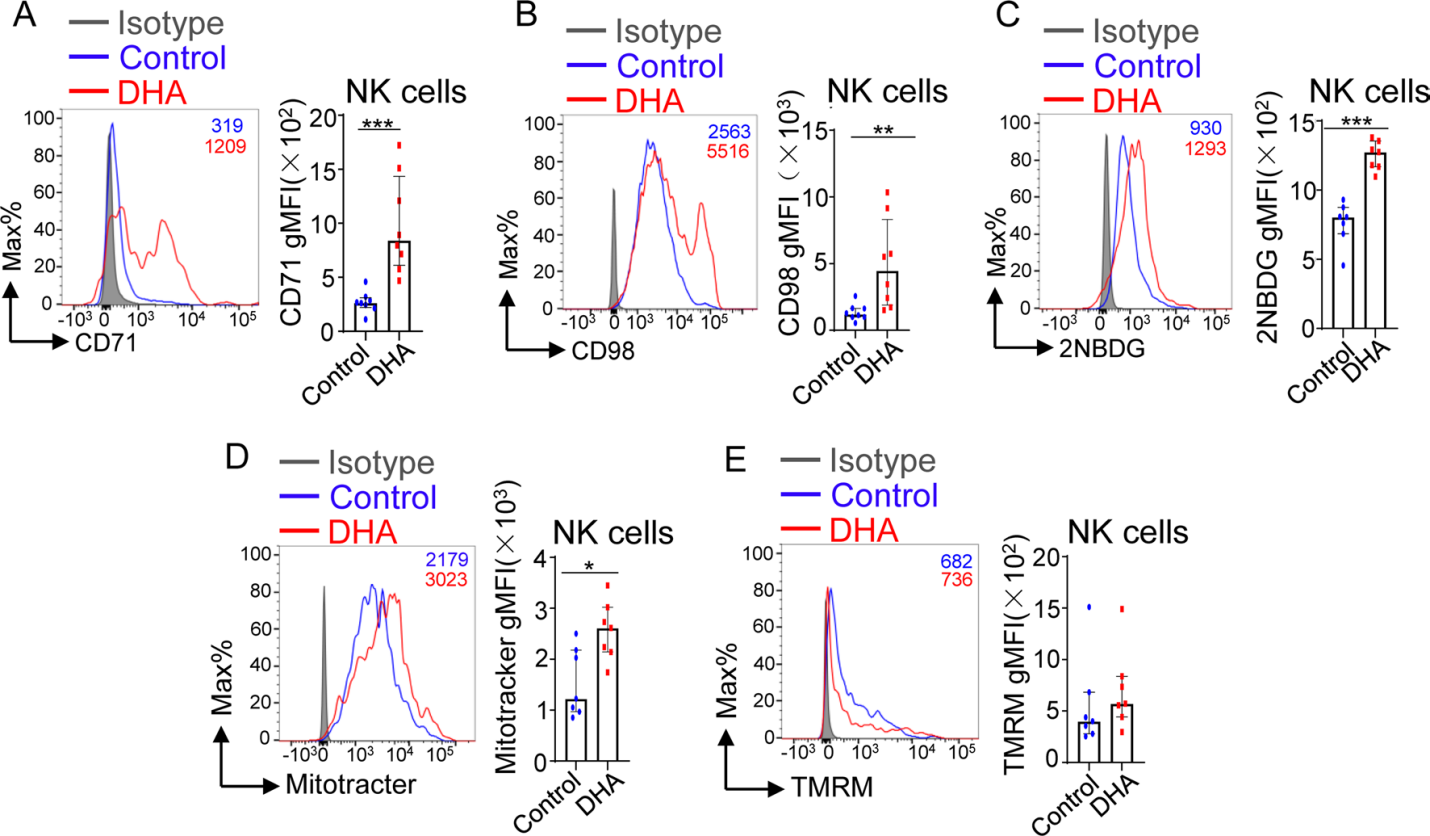
Differences in the cellular metabolic status and mitochondrial activity of splenic NK cells between DHA-fed and control mice at day 7 post-MCMV infection. **A**–**E** Flow cytometric analysis and cumulative results depicting the gMFI of CD71 (**A**) and CD98 (**B**) and the uptake of 2-NBDG (**C**), MitoTracker (**D**) and tetramethylrhodamine, methyl ester (TMRM) (**E**) by splenic NK cells (CD3^−^CD19^−^NK1.1^+^NKp46^+^) from control versus DHA-fed mice at day 7 postinfection. For each experiment, n = 7 pooled from 2 independent experiments. Each symbol represents an individual mouse, and the blue dots and red squares represent control and DHA-enriched diet-fed mice, respectively. Error bars represent interquartile ranges; *p < 0.05; **p < 0.01; ***p < 0.001

To explore the potential mechanism underlying the enhanced NK cell effector function induced by in vivo DHA supplementation, we next tested whether DHA feeding interfered with NK cell metabolic status, a basic process critical for facilitating robust NK cell effector functions [33]. NK cell activation results in increases in the rates of both glycolysis and mitochondrial oxidative phosphorylation (OXPHOS). The expression levels of dedicated transporters, including the transferrin receptor CD71 and amino acid transporter CD98 [33], which control cellular access to nutrients, were substantially increased on the surface of NK cells from the DHA-fed mice compared with control mice at day 7 postinfection (Fig. 4A, B). Glucose uptake, indicated by the fluorescent glucose analog 2-(N-(7-nitrobenz-2-oxa-1,3-diazol-4-yl) amino)-2-deoxyglucose (2-NBDG) [34], was also increased in NK cells from the DHA-fed mice compared with control mice at day 7 postinfection (Fig. 4C).

Mitochondria, the essential hub of metabolic activity, are critical for OXPHOS activity and are the powerhouses of immunity [35]. In general, healthy mitochondria generate a proper membrane potential for the movement of substrates from the cytosol into the mitochondrial matrix for OXPHOS [36]. At day 7 postinfection, we found that splenic NK cells from DHA-fed mice had an increased overall mitochondrial content, as indicated by flow cytometric labeling with MitoTracker, but with no effect on mitochondrial membrane potential, as indicated by TMRM staining (Fig. 4D, E).

Overall, these findings suggest an increased overall rate of cellular metabolism and increased mitochondrial activity in NK cells from DHA-fed mice.

### DHA feeding impaired the cellularity and activation of T cells in the spleen

**Fig. 5:**
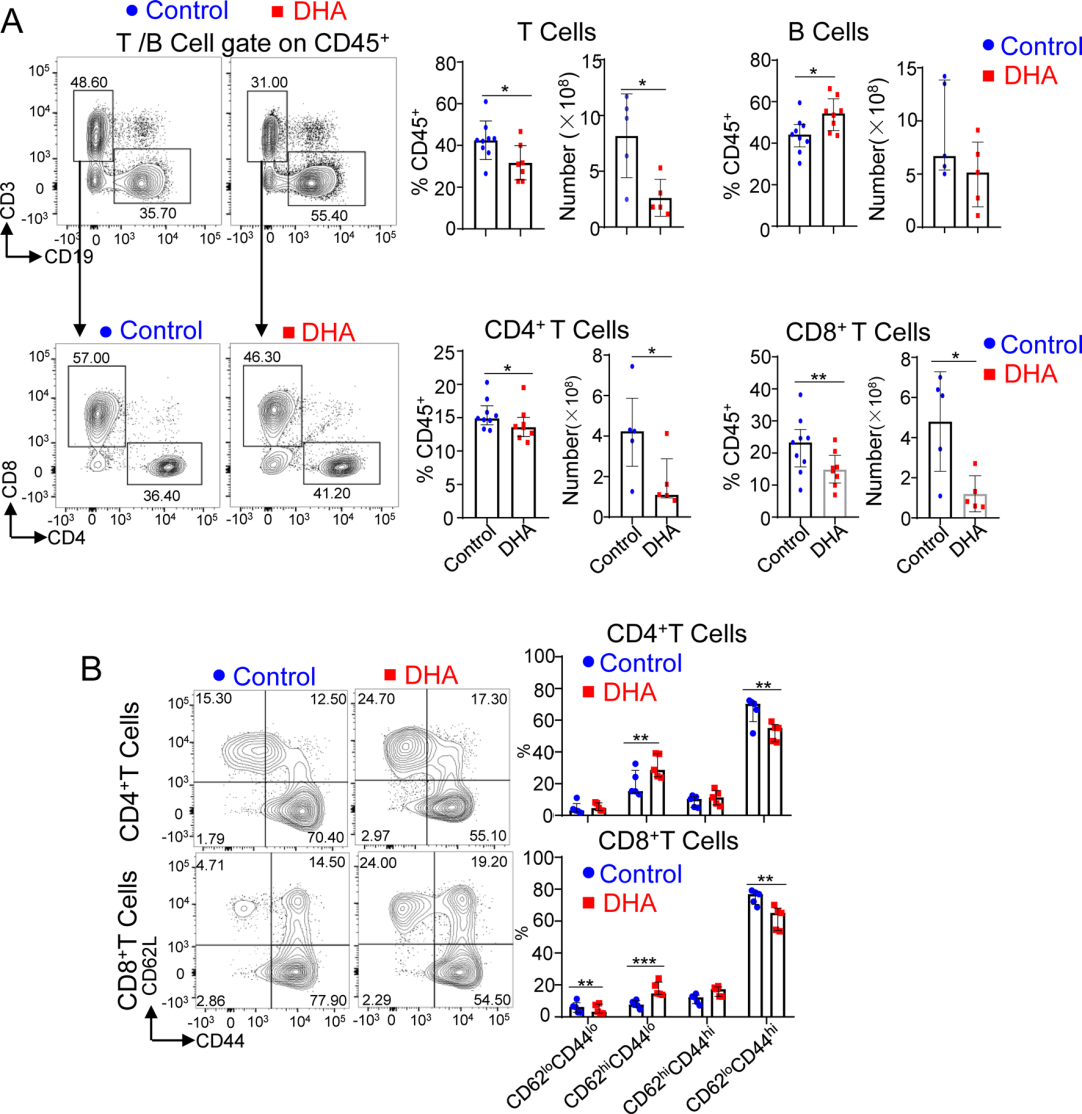
The effects of DHA feeding on the cellularity and activation of T and B cells in the spleen at day 7 postinfection. **A** Flow cytometric analysis and enumeration of total T cells (CD45^+^CD3^−^CD19^−^), CD4^+^ T cells (CD45^+^CD3^+^CD19^−^CD4^+^CD8^−^), CD8^+^ T cells (CD45^+^CD3^+^CD19^−^CD4^−^CD8^+^) and B cells (CD45^+^CD3^−^CD19^+^) in the spleens of control versus DHA-fed mice. **B** Flow cytometric analysis and cumulative data indicating the expression of CD62L and CD44 in CD4^+^ T and CD8^+^ T cells between control and DHA-fed mice. For each experiment, n = 5 to 9 pooled from 2 independent experiments (**A**, **B**). Each symbol represents an individual mouse, and the blue dots and red square represent control and DHA-enriched diet-fed mice, respectively. Error bars represent interquartile ranges; *p < 0.05; **p < 0.01; ***p < 0.001

As the above data showed that DHA feeding inhibited NK cell expansion, we next determined whether DHA feeding would affect adaptive immune response cells, such as T cells and B cells. The data showed that DHA feeding resulted in reduced proportions and numbers of total T cells and subsets of CD4^+^ and CD8^+^ T cells but had no obvious effect on B cell number at day 7 postinfection (Fig. 5A).

To further determine whether DHA feeding affects T cell activation, we measured the ratio of CD62L^hi^CD44^lo^ cells and CD62L^lo^CD44^hi^ cells, which represent naïve and activated T cells, respectively [37], among CD4^+^ and CD8^+^ T cells in both control and DHA-fed mice. We found an increased proportion of CD44^lo^CD62L^hi^ cells but a decreased proportion of CD62L^lo^CD44^hi^cells among both CD4^+^ T cells and CD8^+^ T cells in DHA-fed mice compared with controls (Fig. 5B).

These data demonstrate that DHA feeding also impairs T cell expansion and activation.

### DHA feeding did not obviously affect the mRNA expression of inflammatory mediators in the spleens of MCMV-infected mice

**Fig. 6:**
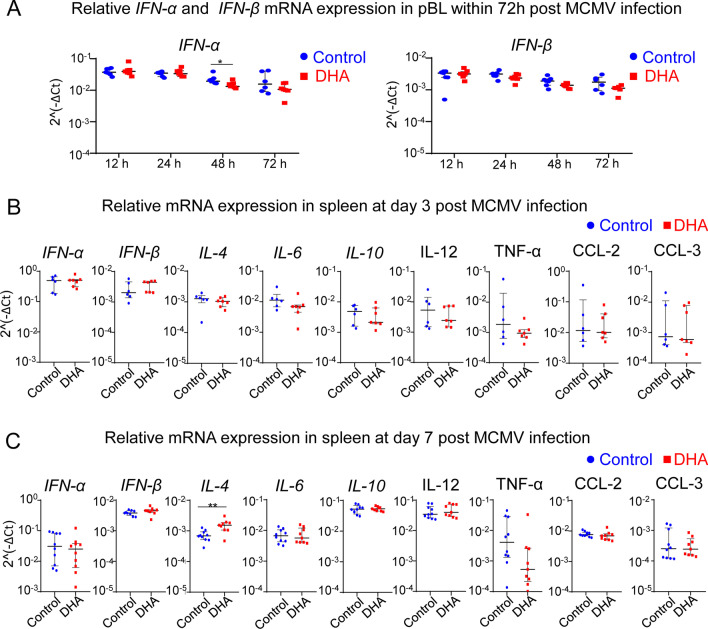
The effects of DHA feeding on the mRNA expression of inflammatory mediators at day 3 and day 7 post-MCMV infection. **A** mRNA levels of *IFN-α* and *IFN-β* in the peripheral blood cells of both control and DHA-fed mice following MCMV infection at 12, 24, 48 and 72 h determined by RT–qPCR. **B**, **C** mRNA levels of the cytokines and chemokines, including *IFN-α*, *IFN-β*, *IL-4*, *IL-6*, *IL-10*, *IL-12*, *TNF-α*, *CCL-2* and *CCL3*, in the spleen of both control and DHA-fed mice at days 3 (**B**) and 7 (**C**) postinfection were determined by RT–qPCR. Each symbol represents an individual mouse, and the blue dots and red square represent control and DHA-enriched diet-fed mice, respectively. For each experiment, n = 5 to 9 pooled from 3 independent experiments (**A**–**C**). Error bars represent interquartile ranges. *p < 0.05; **p < 0.01

Type I IFNs, including IFN-α and IFN-β, are critical for controlling of acute MCMV infection [38, 39], sufficient for the induction of latency immediately upon infection in vitro [40]. Thus, we detected the mRNA levels of both *IFN-α* and *IFN-β* in pBL cells following MCMV infection at 12, 24, 48, and 72 h by RT–qPCR. We found that the mRNA expression of both *IFN-α and IFN-β* was highest at 12 h after MCMV infection in vivo in both DHA-fed and DHA-unfed mice, as previously described [41]. However, only at 48 h after MCMV infection was the mRNA level of *IFN-α* moderately decreased in the DHA-fed mice, compared with the DHA-unfed mice (Fig. 6A).

Coordinated secretion of cytokines and chemokines occurs in the target organ during MCMV infection [38]. To explore whether DHA feeding could influence the expression of various inflammatory mediators, the mRNA levels of these inflammatory mediators in splenic tissue were determined at both days 3 and 7 post-MCMV infection. Our data showed that the mRNA levels of *IFN-α, IFN-β, IL-4, IL-6, IL-10, IL-12, CCL-2* and *CCL-3* in the spleen were comparable between DHA-fed and control mice at day 3 post-MCMV infection (Fig. 6B). At day 7 post-MCMV infection, only the mRNA levels of *IL-4* were moderately increased in the spleens of DHA-fed mice compared with controls (Fig. 6C).

Overall, these data suggest that DHA feeding had a minor effect on the levels of inflammatory cytokines at the early stage of MCMV infection.

## Discussion

The effect of dietary ω-3 PUFA supplementation on the body’s antiviral capacity is still under debate. A recent updated meta-analysis revealed that clinical omega-3 fatty acid supplementation is associated with favorable outcomes in patients with sepsis [10]. Additionally, the results of a recent pilot study of 100 patients infected with SARS-CoV-2 suggested that higher levels of two major ω-3 PUFAs, DHA and EPA, are negatively correlated with the risk of COVID-19 mortality [42]. In the current study, we found that supplementation with DHA, the main component of ω-3 PUFAs, led to a reduced acute response to MCMV infection, as indicated by increases in weight loss and MCMV DNA load at day 7 postinfection (i.e., the early stage). However, DHA feeding did not affect the later stage of MCMV clearance in C57BL/6 mice, as indicated by a lack of difference in viral load in the salivary glands at day 14 postinfection. Additionally, the pathological changes in both the spleen and liver and the mRNA levels of inflammatory mediators in the spleen were almost comparable between DHA-fed and control mice at day 3 and day 7 postinfection. These findings suggest that DHA supplementation does not affect the end of MCMV clearance.

The results of one previous double-blind, placebo-controlled study showed that daily consumption of 1 g ω-3 PUFA for six months reduces CD3^−^CD16^+^CD56^+^ NK cell numbers in human pBL leukocytes, which was inversely, correlated with the levels of DHA and EPA in erythrocytes [29]. Consistently, our data revealed that mice fed DHA for 3 weeks showed a reduced NK cells frequency in the pBL at a steady state. However, DHA feeding did not affect NK cell maturation or Ly49H^+^ NK cell frequency in the absence of MCMV infection. Interestingly, at day 7 postinfection, DHA-fed mice further exhibited inhibited NK cell maturation and Ly49H^+^ NK cell expansion at this time point. In addition, DHA supplementation also reduced the cell numbers and activation levels of T cells at day 7 postinfection. During influenza virus infection, fish oil feeding also decreased the numbers of NK cells and T lymphocytes in the lungs [9]. Collectively, these data indicate that the reduced NK cell numbers, especially the reduction in terminally differentiated NK cells upon MCMV infection, and reduced T cell activation levels contribute to the reduced acute response to MCMV infection as a result of DHA feeding. The mechanisms of DHA feeding resulting in reduced NK cell numbers at a steady state and impaired NK cell maturation upon MCMV infection warrant further study.

Notably, DHA-fed mice showed increased per cell effector functions in NK cells, indicated by an enhanced capacity for IFN-γ production and direct cytotoxicity, and increased expression of the cytotoxicity-related molecule GZMB on NK cells at day 7 post-MCMV infection. NK cell-derived IFN-γ can directly induce CD4^+^ T cell differentiation into type I T helper cells, facilitating the control of bacterial or virus infection [18]. NK cell-derived IFN-γ enhances direct NK-dendritic cell interactions, leading to the upregulation of costimulatory molecules on DCs, resulting in enhanced CD8^+^ T cell effector function. Thus, the enhanced per-cell effector function in NK cells may at least partly compensate for the reduced total NK cell numbers, inhibited maturation and Ly49^+^ NK cells in the subsequent clearance of MCMV-infected cells at the late stage of MCMV infection.

The increase in per-cell NK cell effector function by DHA feeding could be explained by metabolic reprogramming, a key issue involved in regulating NK cell activation and functional maintenance [33]. Our data showed that the uptake of NK cell nutrients, indicated by the enhanced uptake of the glucose analog 2-NBDG and improved expression of the nutrient receptors CD71 and CD98, was enhanced by DHA supplementation. The mitochondrial mass of NK cells in the mice fed DHA was also significantly improved after MCMV infection compared with that in the control mice. This finding might indicate the direct effect of DHA or its derivatives, such as resolvin D1 (RvD1) produced by 15-LOX and 5-LOX and MCTR1 catalyzed by 12-LOX, on mitochondria. The consumption of DHA or other ω-3 PUFAs could remodel the mitochondrial phospholipidome and target mitochondrial enzymatic activity. For example, DHA acts as an agonist of PPARγ, which can promote mitochondrial biogenesis and induce the expression of genes encoding several key mitochondrial enzymes within mitochondria [43]. Dietary supplementation with fish oil for MCMV infection promoted mitochondrial biosynthesis in the liver cells of male C57BL/6 mice [44]. Both RvD1 and MCTR1 have been reported to improve mitochondrial biogenesis and function induced by multiple adverse factors, such as inflammation or high sugar levels [45–47].

## Conclusions

In summary, DHA supplementation reduced NK cell numbers at a steady state and impaired maturation upon MCMV infection, which may play an important role in the inhibited early response to MCMV infection. However, DHA feeding preserves NK cell effector functions by improving mitochondrial activity, guaranteeing sufficient subsequent MCMV clearance in the later stage of infection. Therefore, DHA supplementation does not affect the end of MCMV clearance, and it is still acceptable to use DHA supplementation for chronic disease prevention in the context of MCMV infection.

## Methods

### Animals preparation

Five-week-old male wild-type (WT) C57BL/6J mice were purchased from Hunan Sja Laboratory Animal Co., Ltd. (Changsha, Hunan, China). All mice were housed under specific pathogen-free conditions at the Hunan Children’s Hospital Animal Facility on a 12-h light/dark schedule with free access to food and water. All animal procedures and protocols were approved by the Animal Ethics Committee of Hunan Children’s Hospital and followed the guidelines of the Institutional Animal Care and Use Committees of Hunan Children’s Hospital (Changsha, Hunan, China).

### Virus stock preparation

The MCMV strain Smith (VR-1399) was a kind gift from the College of Life Sciences, Hunan Normal University (Changsha, Hunan, China). Stocks of MCMV Smith strain salivary gland extracts were prepared as previously described [48]. The viral titer is expressed in plaque-forming units (PFU)/mL. BALB/c mice were injected intraperitoneally with 5 × 10^3^ PFU virus particles and euthanized 2 weeks later. All mice were sacrificed by cervical dislocation under anesthesia with 2% pentobarbital sodium. The salivary glands were collected and homogenized to obtain initial salivary gland-derived MCMV, and these steps were then repeated at least 3 times to obtain more virulent virus. PFUs were quantitated by a simple plaque-forming cell assay on 3T3 fibroblasts provided by Dr. Chen Ze (College of Life Sciences, Hunan Normal University, Changsha, Hunan, China) as previously described [49].

### DHA diet and CMV infection

Five-week-old male C57BL/6J mice consumed a control diet (D200208, Research Diets) or a diet containing DHA at physiological levels (2.48% DHA, D201124, Research Diets) ad libitum for 3 weeks (the ingredients of the diets are listed in Table 1). Following 3 weeks of dietary treatment, the mice were infected with MCMV by intraperitoneal injection (3 × 10^4^ PFU, diluted in 200 μL of PBS). The mice were weighed following infection, and the percent weight loss at days 0, 3, 7, 9, 14 and 21 after MCMV infection was calculated by comparison with the starting weight. Mice were subjected to dislocation under anesthesia by 2% pentobarbital sodium at days 3, 7 or 21 after MCMV infection for subsequent studies.

**Table 1:** Composition of experimental diets

Ingredient	Control		DHA	
	g/100 g	kcal/100 g	g/100 g	kcal/100 g
Caisein	20.000	80.000	20.000	80.000
DL-Methionine	0.300	1.200	0.300	1.200
Dyetrose	12.000	48.000	12.000	48.000
Cornstarch	51.800	207.200	51.800	207.200
Soybean oil	6.200	55.800	3.720	33.480
DHA	0.000	0.000	2.480	22.320
Cellulose	5.000	0.000	5.000	0.000
Mineral mix #200,000	3.500	0.000	3.500	0.000
Vitamin mix #300,050	1.000	4.000	1.000	4.000
Choline bitartrate	0.200	0.000	0.200	0.000
Red dye	0.005	0.000	0.005	0.000

### Quantification of MCMV *Ie-1* DNA levels in the liver and spleen by real-time quantitative PCR (qPCR)

Ten milligrams of fresh liver and spleen tissues were cut into small pieces and placed in a 1.5-ml microcentrifuge tube. DNeasy Blood & Tissue Kits (QIAGEN, 69504) were used to rapidly purify of the total DNA. The DNA concentration was tested by biodrop (Biodrop uLite PC) for each sample. Real-time qPCR was performed using Bestar® SYBR Green qPCR Master Mix (DBI Bioscience, San Diego, CA, United States) with a Roche LightCycler® 480 II. A standard curve was created by comparing Cq values to 10 serial dilutions of the *Ie1* plasmid with R^2^ = 0.9953 (Additional file 1: Fig. S3). The number of copies (*Ie-1*) was calculated by comparing the Cq values to the standard curve and dividing by the concentration. The primer sequences specific for the immediate early gene (*Ie-1*) used for qPCR were designed by Primer Bank and are as follows: MCMV *Ie-1* Forward, 5′-GAGTCTGGAACCGAAACCGT-3′ Reverse, 5′-GTCGCTGTTATCATTCCCCAC-3′ [25]. The PCR reaction mixture was adjusted to 10 μL, 5 μL 2 × SYBR Green pro taq HS Premix, 0.2 μM primers, 1 μL DNA extraction, 3.6 μL RNase free water. The amplification program was as follows: 30 s at 95 °C for denaturation, 40 cycles that consisted of a step at 95 °C for 5 s followed by 60 °C for 30 s for annealing and 30 s at 72 °C for extension.

### Pathological evaluation of spleen and liver tissues

The liver and spleen were removed at day 7 postinfection and fixed with 10% phosphate-buffered formalin, paraffin-embedded, cut into 4-µm sections, and stained with hematoxylin and eosin, as described previously [50]. The pathological score for each slide was calculated by an anatomopathologist blinded to the sample information, as previously described [25]. Briefly, for grading of spleen inflammation, scores of 0, 1, 2, 3, and 4 were assigned, indicating normal, mild (multifocal pyogranulomas in marginal zones), moderate (locally coalescing pyogranulomas in marginal zones with small necrotic foci), substantial (large and coalescing pyogranulomas throughout the splenic parenchyma with extensive necrotic foci, the periarteriolar lymphoid sheath preserved), and severe (extensive necrotic and pyogranulomatous foci, the periarteriolar lymphoid sheath partially replaced by necrotic and granulomatous inflammation), respectively. For liver inflammation grading, scores of 0, 1, 2, 3, and 4 were assigned, indicating normal, mild (multifocal pyogranulomatous hepatitis with scattered single necrotic hepatocytes); moderate (multifocal to coalescing necrotic and pyogranulomatous hepatitis with intranuclear inclusions in hepatocytes); and substantial (coalescing necrotic and pyogranulomatous hepatitis with intranuclear inclusions in hepatocytes), respectively.

### Preparation of single-cell suspensions and counts

Single-cell suspensions were prepared from the BM, spleen, pLNs, liver and pBL as previously described. pBL was treated with erythrocyte lysate. The BM, spleens, pLNs and livers were ground and passed through a 40-μm nylon filter. The obtained liver cells were resuspended in 40% Percoll in RPMI 1640 medium containing 5% FBS and then centrifuged (2000 rpm, 4 °C, 5 min). Cell pellets were resuspended in RPMI 1640 medium containing 5% FBS. The cells from each tissue were counted with an automated cell counter (Countstar IC1000).

### Flow cytometry

All antibodies purchased for flow cytometry are listed in Table 2. Standard protocols were followed for flow cytometry, as previously described [51]. All flow cytometry experiments were carried out on a BD LSRFortessa™ cell analyzer, and data were analyzed with FlowJo software.

**Table 2:** Antibodies for flow cytometry

Antibody	Clone	Source	Identifier
Anti-mouse-NK1.1	PK136	BioLegend	Cat#108708 Cat#108753
Anti-mouse NKp46	29A1.4	BioLegend	Cat#137618 Cat#137608
Anti-mouse CD11b	M1/70	BioLegend	Cat#101228
Anti-mouse KLRG1	2F11KLRG1	BioLegend	Cat#138414
Anti-mouse CD62L	MEL-14	BioLegend	Cat#104438
Anti-mouse CD45	30-F11	BioLegend	Cat#103154
Anti-mouse CD3	17A2	BioLegend	Cat#100216 Cat#100320
Anti-mouse CD19	605	BioLegend	Cat#115520 Cat#115528
Anti-mouse GZMB	GB11	BioLegend	Cat#515406
Anti-mouse perforin	S16009A	BioLegend	Cat#154306
Anti-mouse CD27	LG 3A10	eBioscience	Cat#124229
Anti- mouse CD16/32	2.4G2	BD Biosciences	Cat#553141
Anti-mouse Ly49H	3D10	BD	Cat#744262
Anti-mouse CD4	RM4-5	BD	Cat#550954
Anti-mouse CD8a	53–6.7	BD/Biolegend	Cat#553030 Cat#563898
Anti-mouse CD71	C2	BD	Cat#553266
Anti-mouse IFN-γ	XMG1.2	BD	Cat#554411
Anti-mouse CD98	RL388	Invitrogen	Lot#2074373
Anti-mouse CD44	IM7	BioLegend	Cat#103044

Briefly, to detect surface markers, cells were stained with antibodies in staining buffer (phosphate-buffered saline (PBS) containing 2% mouse serum, 2% horse serum, and anti-CD16/CD32 blocking antibodies) in the dark for 15 min at room temperature. For intracellular IFN-γ staining, cells were stimulated with phorbol 12-myristate 13-acetate (PMA) and ionomycin (eBioscience) plus BD Golgi Plug™ and Golgi Stop™ protein transport inhibitor (BD Biosciences) for 4 h, and the cells were then stained with reagent from a Fixation/Permeabilization Solution Kit (BD Biosciences) following the manufacturer’s instructions.

For intracellular proteins (including granzyme B and perforin), cells were stained with surface antibodies, permeabilized with reagent from a Foxp3/Transcription Factor Staining Buffer Set Kit (eBioscience), and then stained with anti-granzyme B and anti-perforin antibody or isotype-matched control antibody.

To determine the direct cytotoxicity of NK cells in both DHA-fed/unfed mice after MCMV infection, purified NK cells were sorted (purity ≥ 95.0%) from the spleen of both DHA-fed and control mice at day 7 after MCMV infection. YAC-1 cells were labeled with CTV (5 µM, Thermo Fisher Scientific, Waltham, United States) according to the manufacturer’s instructions. After washing, 5000 labeled YAC-1 cells were cocultured with the above purified NK cells for 6 h at an effector-to-target (E:T) ratio of 16:1 [52]. Target NIH-3T3 cells were infected with 3 PFU/cell MCMV [53], and 16 h later, these cells were labeled with CTV followed by coculture with purified NK cells as performed for the YAC-1 cell coculture. Specific lysis of target cells was assessed by staining each sample with 1 µg/ml 7-AAD (BD) and measuring by an LSRFortessa™ cell analyzer. As a control, samples containing only target cells were stained with 7-AAD to measure spontaneous cell death. NK cell cytotoxicity was calculated by the following formula, as previously described: [(% CTV^+^ 7-AAD^+^ cell specific lysis − %CTV^+^ 7-AAD^+^ cell spontaneous lysis)/(100 − % CTV^+^ 7-AAD^+^ cell spontaneous lysis)] × 100 [53–55]. All groups were tested in triplicate.

To determine the glucose uptake capacity of NK cells, the cells were cultured with prewarmed (37 °C) RPMI 1640 medium (Life Technologies) containing 100 μM 2-(N-(7-nitro-benz-2-oxa-1,3-diazol-4-yl) amino)-2-deoxyglucose (2-NBDG, a fluorescent glucose analog) (Invitrogen) for 10 min at 37 °C in the dark.

To determine mitochondrial activity, splenic cells were cultured (37 °C, 30 min) with prewarmed (37 °C) RPMI 1640 medium containing 20 nM MitoTracker® Green FM (Invitrogen) or tetramethylrhodamine ethyl ester (TMRM) (Invitrogen) in the dark.

### Quantitation of spleen mRNA cytokine and chemokine levels

Mouse peripheral blood cells were collected 12 h, 24 h, 48 h and 72 h after MCMV infection and treated with red blood cell lysis buffer before RNA extraction with the total RNA purification Micro Kit (NORGEN Cat.35300). The spleens were removed from DHA-fed and control mice at day 7 postinfection. The TRIzol method was used to isolate total RNA, and reverse transcription (RT) was conducted with Evo M-MLV RT Premix for qPCR (Accurate Biology). The mRNA levels of *IFN-α*, *IFN-β*, *IL-4*, *IL-6*, *IL-10*, *IL-12*, *TNF-α*, monocyte chemotactic protein-1 (*MCP-1*, also referred to as *CCL-2*) and macrophage inflammatory protein-1-a (*MIP-1α*, also called *CCL3*) were measured using real-time RT-qPCR. The primer sequences we used for *IFN-α* could simultaneously detect the mRNA expression of four subtype of *IFN-α* in mouse, including *IFN-α1*, *IFN-α2*, *IFN-α7*, and *IFN-α11* [40, 56]. The PCR reaction mixture was adjusted to 10 μL, 5 μL 2 × SYBR Green pro taq HS Premix, 0.2 μM primers, 1 μL DNA extraction, 3.6 μL RNase free water. The amplification program was: 30 s at 95 °C for denaturation, 40 cycles that consists of a step at 95 °C for 5 s followed by 60 °C for 30 s for annealing and 30 s at 72 °C for extension. The Ct values were normalized to the internal control. RNA levels were calculated by the following expression: (2^−△Ct^). The primer pairs used for RT-qPCR are listed in Table 3.

**Table 3:** Primers of cytokine and chemokine

Primer	Sequence (5′-3′)	References
TNF-α-F	TGCCTATGTCTCAGCCTCTTC	[57]
TNF-α-R	GGTCTGGGCCATAGAACTGA	[57]
IL-4-F	GGCATTTTGAACGAGGTCACA	[58]
IL-4-R	AGGACGTTTGGCACATCCA	[58]
IL-6-F	TGTGCAATGGCAATTCTGAT	[57]
IL-6-R	GGTACTCCAGAAGACCAGAGGA	[57]
IL-10-F	GCTCTTACTGACTGGCATGAG	[59]
IL-10-R	CGCAGCTCTAGGAGCATGTG	[59]
IL-12-F	GAGGTGGACTGGACTCCC	[60]
IL-12-R	GCAGGGAACACATGCCCA	[60]
IFN-α-F	TGTCTGATGCAGCAGGTGG	[56]
IFN-α-R	AAGACAGGGCTCTCCAGAC	[56]
IFN-β-F	CTGGCTTCCATCATGAACAA	[61]
IFN-β-R	CATTTCCGAATGTTCGTCCT	[61]
MCP-1-F	TTAAAAACCTGGATCGGAACCAA	[62]
MCP-1-R	GCATTAGCTTCAGATTTACGGG	[62]
MIP-1α-F	ATGAAGGTCTCCACCACTGCCCTTG	[63]
MIP-1α-R	GGCATTCAGTTCCAGGTCAGTGAT	[63]
Ie-1-F	GAGTCTGGAACCGAAACCGT	[25]
Ie-1-R	GTCGCTGTTATCATTCCCCAC	[25]
GAPDH-F	AGGTCGGTGTGAACGGATTTG	[64]
GAPDH-R	TGTAGACCATGTAGTTGAGGTCA	[64]

### Statistical analysis

The sample size was 3–9 per group, and two or three independent experiments were performed for each experiment. Statistical analysis was carried out using SPSS 23.0 (Chicago, IL, United States). The data in this study are expressed as the Median with an interquartile range. If the data were normally distributed, an unpaired two-tailed Student’s *t*-test was used to analyze the differences between two independent groups. Otherwise, a two-tailed Mann–Whitney *U* test was used to analyze the differences, and differences with a *p-*value ≤ 0.05 were considered significant. All graphs were generated by GraphPad Prism 8.0 (GraphPad Software Inc., La Jolla, CA, United States).

## Supplementary Information

**Additional file 1.** Supplementary figure 1–3 and supplementary figure legend 1–3.

## Data Availability

All data are included in the manuscript. The datasets analyzed in the current study are available from the corresponding author on reasonable request.

## Abbreviations

DHA: Docosahexaenoic acid
NK cells: Natural killer cells
CMV: Cytomegalovirus
MCMV: Murine cytomegalovirus
PUFAs: Polyunsaturated fatty acids
EPA: Eicosapentaenoic acid
WT: Wild-type
PFU: Plaque-forming units
qPCR: Real-time quantitative PCR
RT: Reverse transcription
BM: Bone marrow
pLNs: Peripheral lymph nodes
pBL: Peripheral blood
PBS: Phosphate-buffered saline
2-NBDG: 2-(N-(7-nitro-benz-2-oxa-1,3-diazol-4-yl) amino)-2-deoxyglucose
TMRM: Tetramethylrhodamine, methyl ester
OXPHOS: Oxidative phosphorylation
RvD1: Resolvin D1
gMFI: Geometric mean fluorescence intensity
