# Supplementary material for: Docosahexaenoic acid supplementation represses the early immune response against murine cytomegalovirus but enhances NK cell effector function
Source: BMC Immunol. 2022 Apr 19;23:17. doi: 10.1186/s12865-022-00492-6 (PMC9017742; doi:10.1186/s12865-022-00492-6)
Supplement: Supplementary file 1 — Additional file 1. Supplementary figure 1–3 and supplementary figure legend 1–3. [file 12865_2022_492_MOESM1_ESM.docx]

**Docosahexaenoic acid supplementation represses the early immune response against murine cytomegalovirus but enhances NK cell effector function**

Shuting Wu^1,2*^, Shanshan Wang^3^, Lili Wang^1,2*^, Hongyan Peng^1,2^, Shuju Zhang^1,2^, Qinglan Yang^1,2^, Minghui Huang^1,2^, Yana Li^1,2^, Shuzhen Guan^1,2^, Wenjuan Jiang^1,2^, Zhaohui Zhang^4^, Qinghua Bi^4^, Liping Li^1,2^, Yuan Gao^5^, Peiwen Xiong^1,2^, Zhaoyang Zhong^6^, Bo Xu^7#^, Yafei Deng^1,2#^, Youcai Deng^4#^

**Additional file**

**Additional file figures and figure legends**


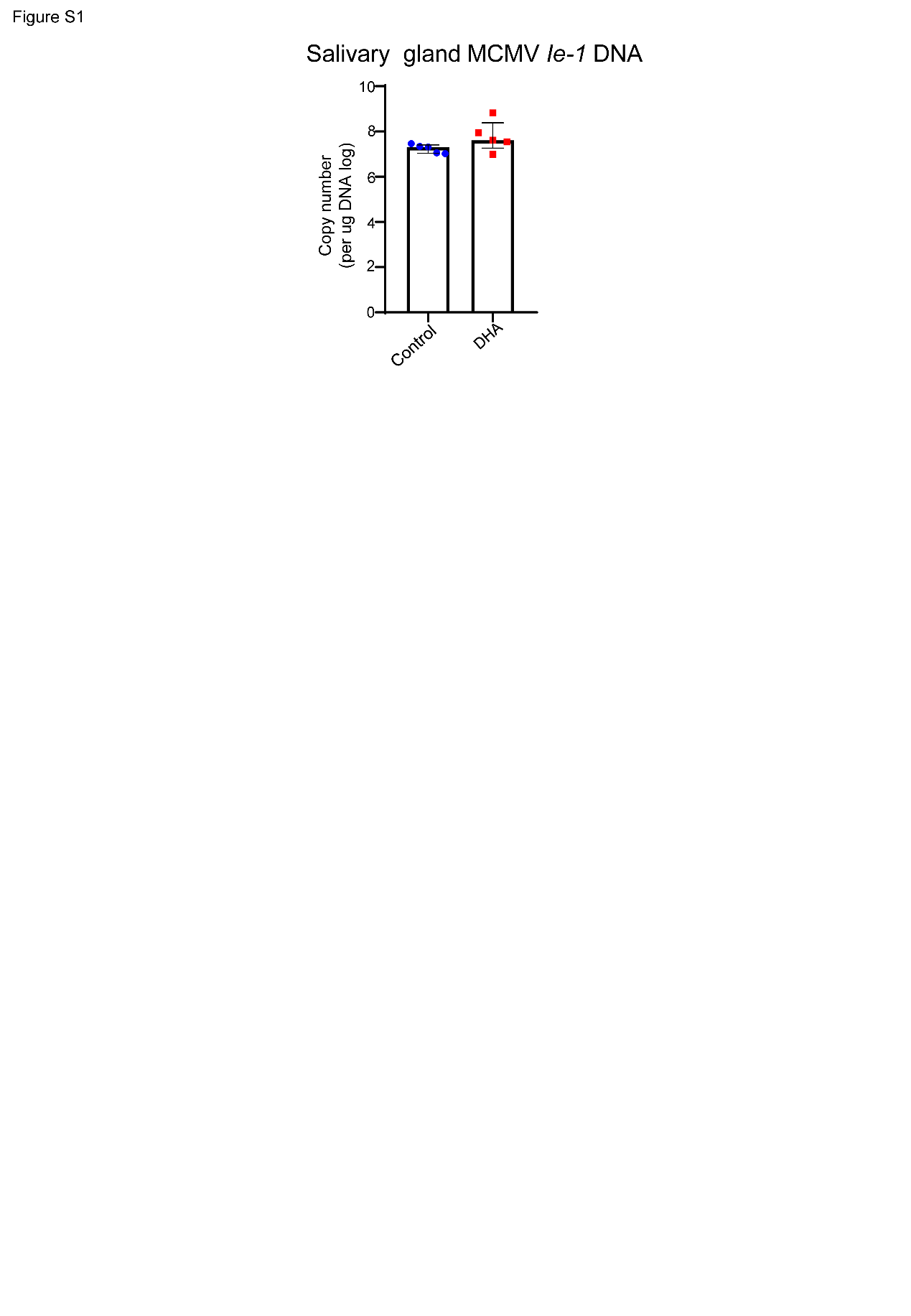


**Additional file 1: Figure S1.** The effect of DHA feeding on MCMV clearance in the salivary glands at day 14 post- MCMV infection. The DNA levels of MCMV *Ie-1* in the salivary glands of both control and DHA-fed mice at day 14 post-MCMV infection were determined by qPCR. n = 5 pooled from 2 independent experiments. Error bars represent interquartile ranges.


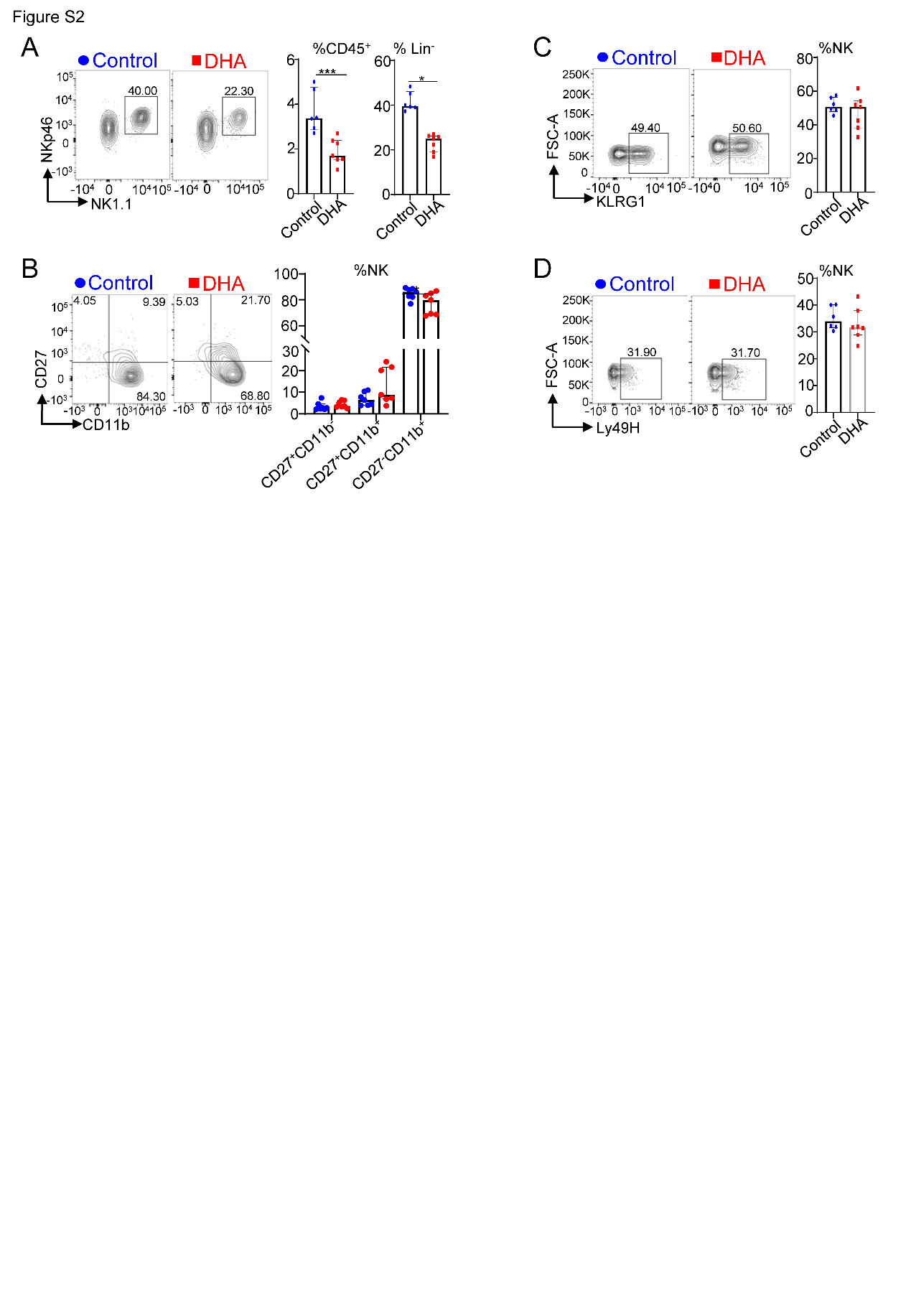


**Additional file 1: Figure S2.** The effect of 3 weeks of DHA feeding on NK cell numbers, maturation and Ly49H^+^ NK cell expansion the day before MCMV infection. **A** Flow cytometric analysis of NK cells (CD45^+^CD3^−^CD19^−^ NK1.1^+^NKp46^+^) in the peripheral blood (pBL) of control versus DHA-fed mice. **B** Flow cytometric analysis and cumulative frequencies of subpopulations of NK cell (CD3^−^CD19^−^NK1.1^+^NKp46^+^) subsets based on CD11b and CD27 expression in the pBL of control versus DHA-fed mice. **C-D** Flow cytometric analysis and enumeration of the KLRG1^+^ (**C**) and Ly49H^+^ (**D**) subsets of NK cells (CD3^−^CD19^−^NK1.1^+^NKp46^+^) in the peripheral blood of control versus DHA-fed mice. For each experiment, n = 6 to 7 pooled from 2 independent experiments (**A-D**). Each symbol represents an individual mouse, and the blue dots and red square represent control and DHA-enriched diet-fed mice, respectively. Error bars represent interquartile ranges; *, *p* < 0.05; **, p < 0.01; ***, *p* < 0.001.


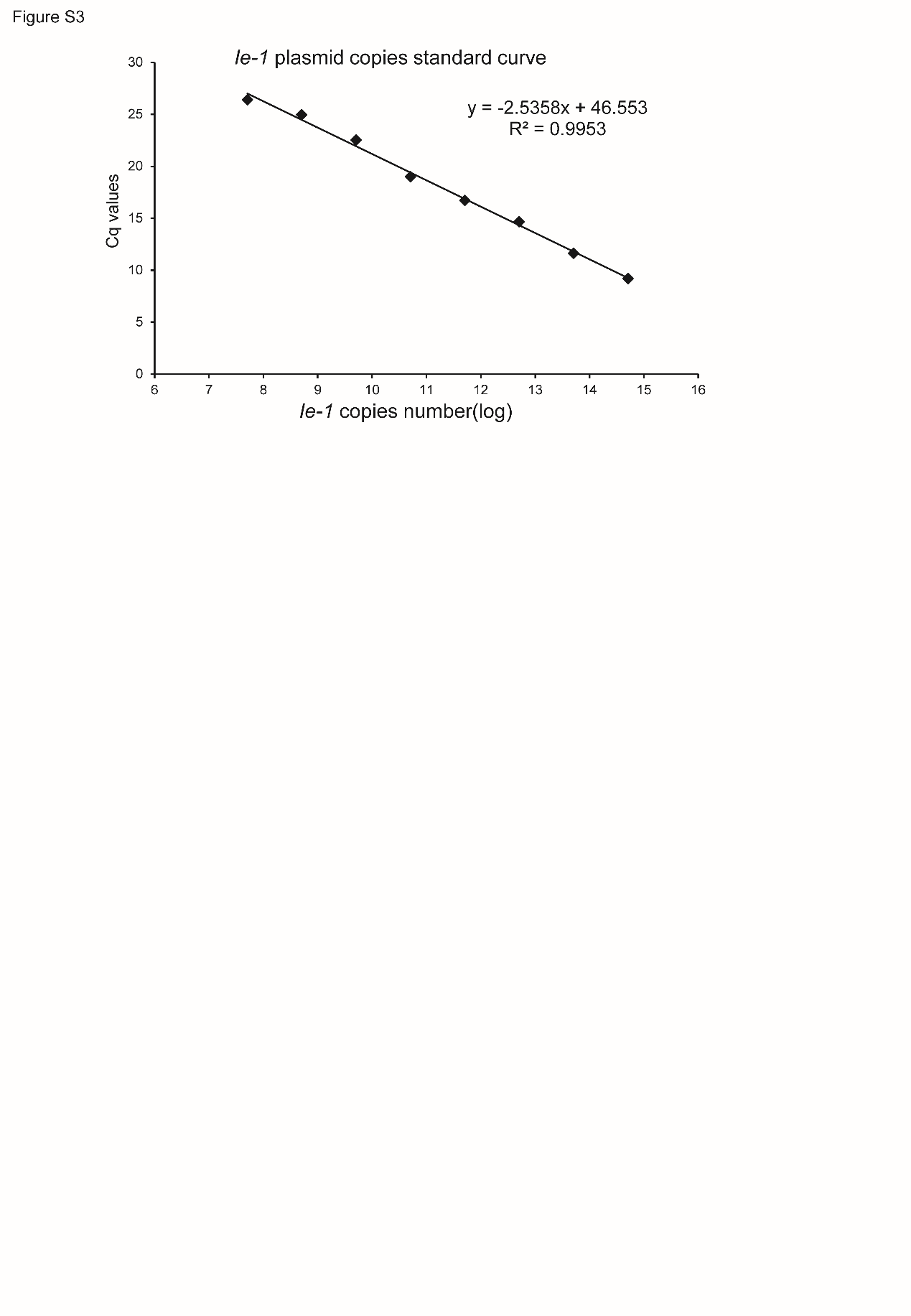


**Additional file 1: Figure S3.** Standard curve of *Ie1* generated for real-time qPCR for MCMV detection and quantification in mouse tissue samples. The input levels of the MCMV *Ie-1* copy numbers on the X-axis and the Ct values on the Y-axis are shown on the standard curve. Linear regression equations and R^2^ are calculated by Office 2020 Excel software.
